# Metastatic Malignancy Masquerading as Skin Rash

**DOI:** 10.7759/cureus.97879

**Published:** 2025-11-26

**Authors:** Hareem Fatima

**Affiliations:** 1 Acute Internal Medicine, University Hospital of North Durham, Durham, GBR

**Keywords:** adenocarcionoma of the lung, cancer immunotherapy, idiopathic inflammatory myopathies, malignancy associated rash, painful skin lesions, paraneoplastic syndromes, photosensitive rash, rare skin disease, rash

## Abstract

Dermatomyositis (DM) is a rare autoimmune inflammatory myopathy characterized by specific skin findings and symmetric proximal skeletal muscle weakness. Many of the affected population have an underlying malignancy, which could naturally alter the condition’s prognosis. This is a case of DM driven by adenocarcinoma of the lung, and the challenges this rare phenomenon led to, with regard to management.

A 72-year-old man presented to the hospital with itchy, maculopapular, blanching lesions across the face, neck, upper chest, arms and hands. The condition was associated with peripheral edema and inflammatory erythema, particularly affecting the upper limbs. The patient also had rapid deterioration in limb muscle weakness. Initial investigations revealed significantly raised muscle injury and inflammatory markers. MRI of the arms demonstrated marked oedema involving the subcutaneous soft tissues as well as the muscles of both upper arms, indicative of DM/myositis. The patient was reviewed by the dermatology team, who gave a clinical diagnosis of DM. He received conservative management of the skin symptoms from dermatology, high-dose intravenous methylprednisolone, intravenous immunoglobulins from rheumatology and palliative management of painful rash and limbs. A thorax, abdomen and pelvis CT was also performed because of the association of DM with malignancy and unfortunately revealed a lung lesion, later confirmed to be an adenocarcinoma of lung origin on biopsy. In DM merely associated with cancer, the DM symptoms often precede the cancer diagnosis. In contrast, paraneoplastic DM symptoms generally develop after the cancer is already present, which is what led us to believe this was paraneoplastic in nature.

The patient was reviewed by the respiratory and oncology teams for management of his cancer. This proved to be especially challenging given that his pathology results suggested a response to immunotherapy would be high and durable in nature. However, given the severity of his DM and the level of immunosuppression required, immunotherapy could not be given first line because it would have significantly risked worsening his DM. He was then considered for chemotherapy after long and challenging multidisciplinary team discussions. However, before treatment could be initiated, the patient unfortunately passed away.

Lung cancer causing DM is a rare phenomenon, which makes it difficult to decide how best to treat the patient. Based on his pathology results, this patient’s particular cancer would have shown a good response to immunotherapy. However, this was not possible given the severity of his DM. Prognosis calculation in this situation also proved difficult.

## Introduction

Dermatomyositis (DM) is an idiopathic, inflammatory myopathy characterized by skeletal muscle weakness and skin changes. It is an uncommon condition, with an annual incidence of 0.1-6 per population of 100,000. Adults aged 50-60 years are the peak affected group. Women are affected twice as often as men are [1]. 

The pathognomonic signs of DM are Gottron’s papules, Gottron’s sign and the heliotrope rash [2]. Other skin manifestations comprise of a maculopapular violaceous erythema of the nape of the neck, the shoulders and upper arms (the Shawl sign), which can also affect the upper chest (the ‘V’ sign); photosensitivity; the holster sign (erythema affecting the buttocks, hips and lateral thighs); and poikiloderma (patches of red-brown atrophic skin with prominent dilated blood capillaries) [3].

In addition, DM (as the name implies) also has muscular symptoms. The most commonly affected muscles are the proximal muscles, usually in the upper arms and thighs. Eventually, they may also become tender to touch. 

Diagnosis involves a combination of history and examination findings, various blood tests, an electromyography, biopsy and magnetic resonance imaging (MRI). Blood tests assess the extent of myositis, with tests such as the creatine kinase (CK), lactate dehydrogenase, alanine transaminase and aspartate aminotransferase, and various autoantibodies.

In this patient's case, diagnosis was mainly based on clinical presentation, supported by blood markers of myositis, and confirmed by MRI imaging of the muscles. 

Of all cases of DM, 10-20% are associated with cancer, with the association rising to > 50% if the patient’s age is 65 years or older. It is most commonly associated with breast, lung, pancreas, stomach, colon and ovarian malignancies. Relevant and appropriate testing to detect any possible underlying malignancy is necessary. Malignancy is only considered in adult DM. Clinical features associated with an increased risk include age > 45 at diagnosis, male gender, dysphagia, cutaneous necrosis, cutaneous vasculitis and rapid onset of skin/muscle symptoms [4].

The highest risk of developing cancer in patients with DM occurs within the first year of onset of myositis. Older patients, those with more severe findings on skin/muscle biopsy (cutaneous necrosis, capillary damage and cutaneous leukocytoclastic vasculitis), those with prior history of cancer, and those with treatment resistance are at a higher risk of developing cancer within the DM population [5].

DM can also present as a paraneoplastic syndrome, which is what this case is about. This patient presented with an extensive rash and myopathy, and in several investigations down the line, he was found to have a DM driven by a previously unknown adenocarcinoma of the lung. 

## Case presentation

**Figure 1:**
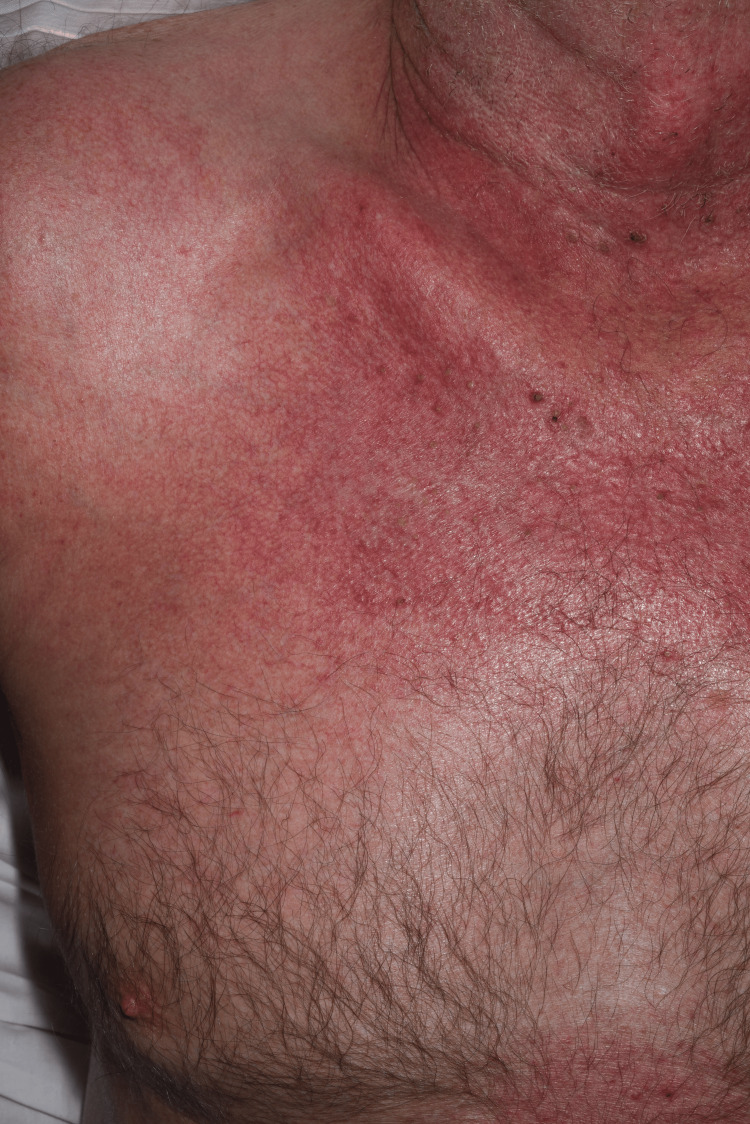
Photo-sensitive distribution of the rash (neck and upper chest)

**Figure 2:**
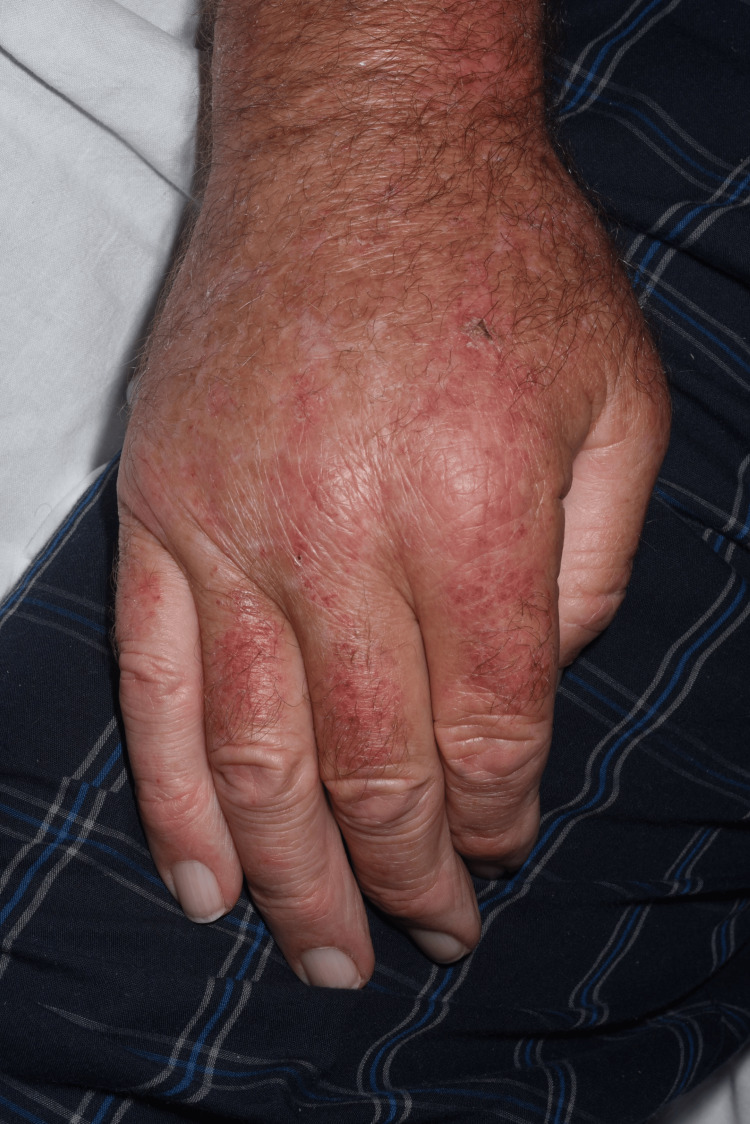
Significantly oedematous hand with characteristic dermatomyositis rash

This was a case of a 72-year-old man of White British ethnic origin. He was previously fit and well and presented to acute medicine due to a new onset, widespread rash. The rash started to appear only within the last few weeks prior to presentation and appeared to be distributed across the face, neck, upper chest, dorsal arms and hands (Figure 1). Marked peripheral oedema and inflammatory erythema, affecting the upper limbs in particular, were associated with the rash (Figure 2). He also had limb muscle weakness, more or less since the start of the rash, which was progressing rapidly.

Significant findings in his initial blood work were a high CRP of 118 mg/L (normal value: < 5 mg/L), erythrocyte sedimentation rate 58 mm/hour (normal range: 2-10 mm/hour) and, most notably, a CK level of 14,394 IU/L (normal range: 59-104 IU/L). He also had a neutrophilia and an alanine transaminase of 185 U/L (normal value: < 40 U/L), both also driven by DM. Multiple specialties were taken on board, starting with dermatology which, based on a clinical diagnosis of DM, commenced treatment conservatively with topical steroids the day of admission and advised rheumatology input.

**Figure 3:**
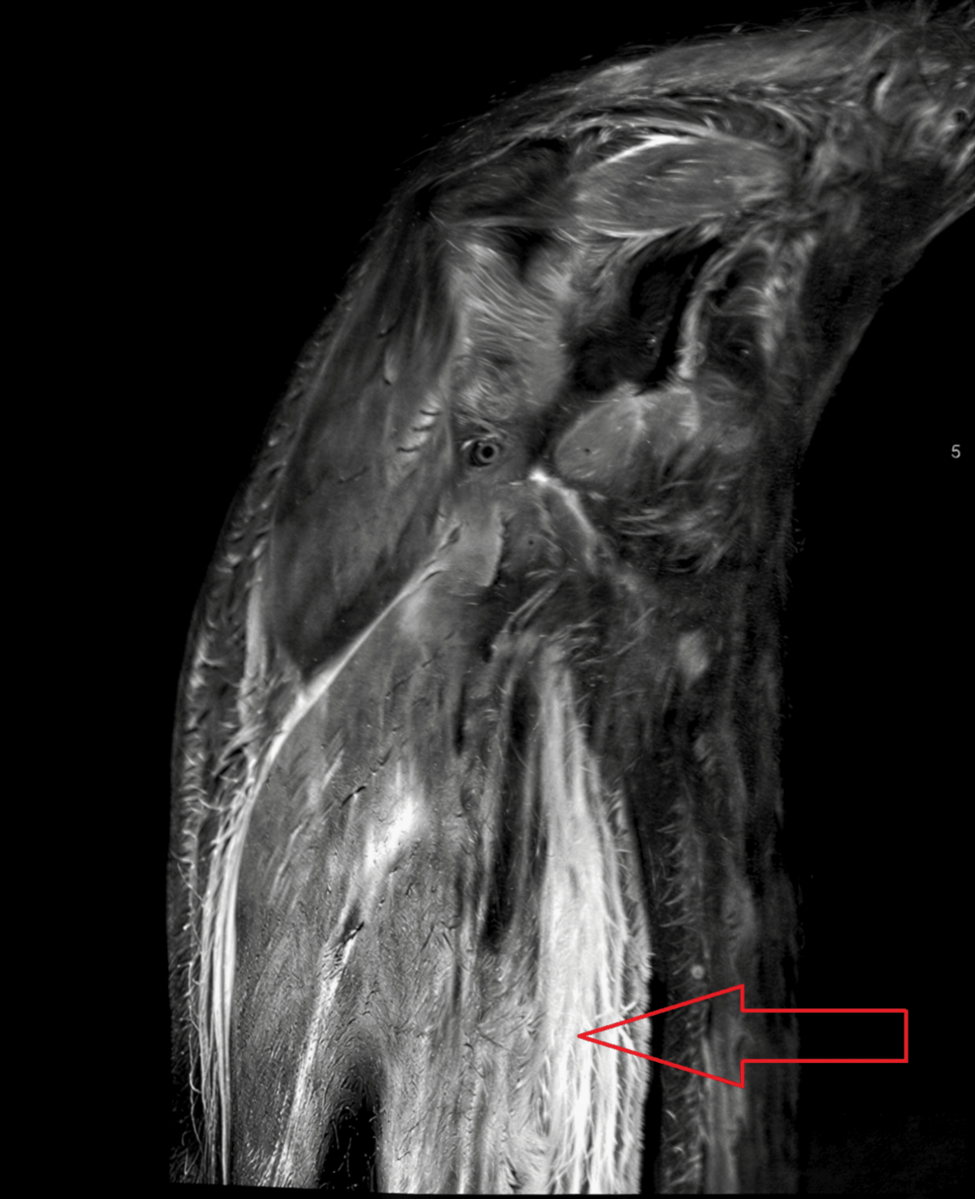
MRI image of the patient's right upper arm, demonstrating changes in keeping with myositis, showing marked involvement of the proximal muscles

On rheumatology advice, an MRI of the arms was done two days after admission, demonstrating marked oedema involving the subcutaneous soft tissues as well as both upper arm muscles, indicative of myositis (Figure 3). 

With this, all of the following diagnostic criteria for DM were met: (a) at least one skin symptom, (b) symmetrical muscle weakness, (c) elevation of serum levels of skeletal muscle-related enzymes and (d) muscle pain [6]. Based on these findings, rheumatology began aggressive treatment with intravenous corticosteroids immediately after the MRI, followed by intravenous immunoglobulin therapy two weeks later. This resulted in improvement in rash and myositis symptoms, as well as a drop in CK levels to 1,126 within one week.

**Figure 4:**
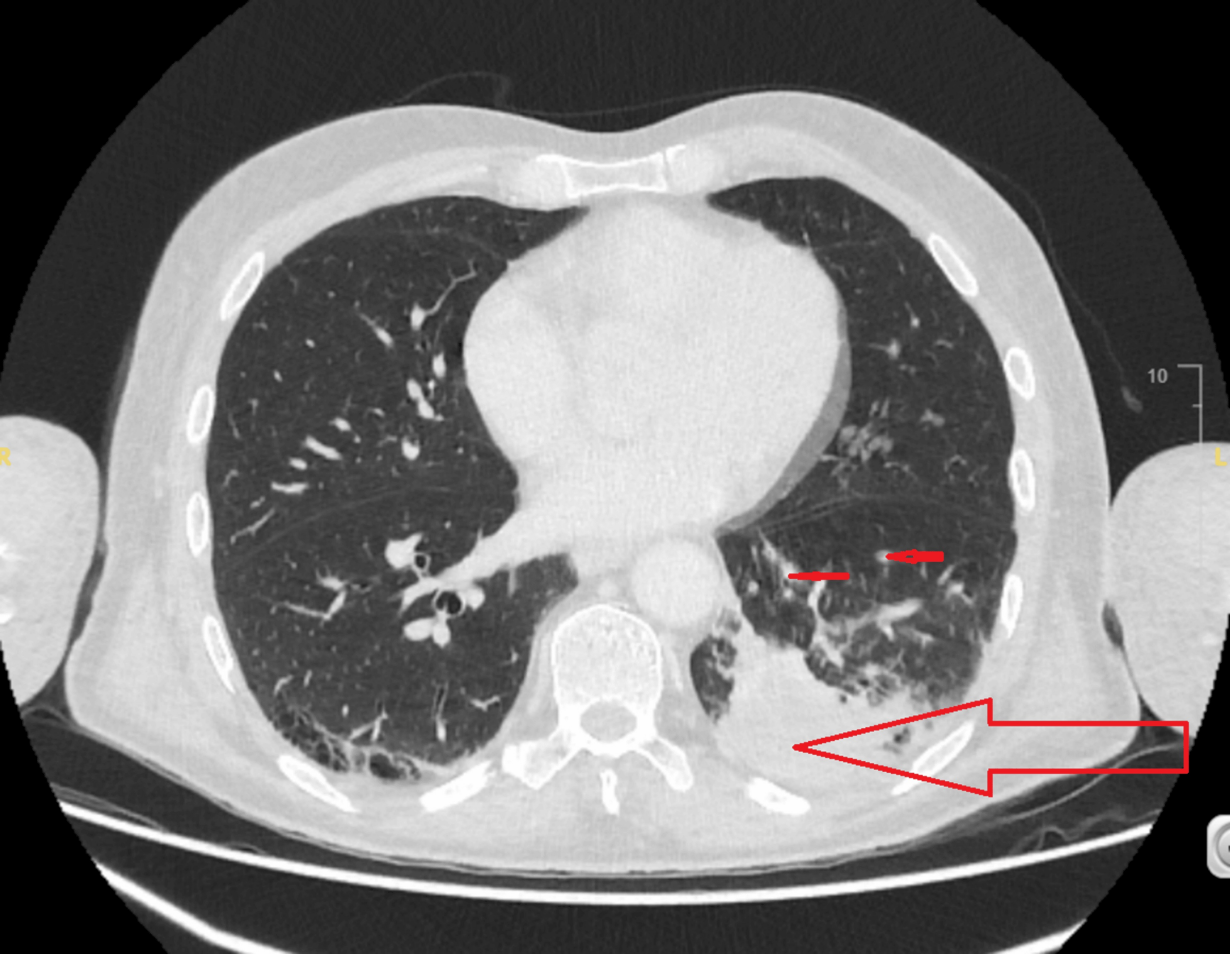
Left lower lobe mass-like consolidation with satellite nodes in a distribution consistent with lung cancer

A thorax, abdomen and pelvis CT was done to detect an underlying malignancy and unfortunately a consolidation collapse opacity in the basal segment of the left lower lobe (LLL). This appearance was attributed to being due to an underlying necrotic mass. In addition, a 1 x 1 cm nodule was detected in the apical segment of the LLL. Multiple left hilar, mediastinal and left supraclavicular nodes were also seen (Figure 4). 

We then proceeded to investigate the supraclavicular lymph node further, and ultrasound-guided biopsy revealed the following: *'Histology shows core and fragments of fibroconnective tissue infiltrated by a non-small cell carcinoma. Immunohistochemistry: TTF1 - positive (strong). P40 - negative. CK 5/6 - negative. The appearances are consistent with a metastatic adenocarcinoma of lung origin.'*

## Discussion

Lung cancer causing DM is a rare phenomenon. When this patient presented, the oncologist mentioned that in the northeast of England, the wider group of oncologists had only seen three other cases in the last eight years. It is equally rare in the literature; we highlight this because how and when best to treat patients is not clear.

After initial treatment, his rash, muscle weakness, and biochemical findings improved. However, the multi-disciplinary team felt that because the cancer had spread outside of his lungs (T2, N3, M0), the disease was incurable and any treatment would be palliative in nature. One oncologist discussed the possibility of transferring the patient to the tertiary oncology centre for initiation of chemotherapy. Within the multi-disciplinary team, a clear consensus could not be reached with regard to starting chemotherapy. It was decided to await the pathology results and rediscuss this in a few days.

One week later, this was revisited with the patient. His full pathology results had not yet come back, but his programmed death-ligand 1 was 60-70% (high), which suggested that a response to immunotherapy would be high and durable in nature. One in three patients treated with immunotherapy first line live for five years. However, given the severity of the patient’s DM and the level of immunosuppression required, immunotherapy could not be given to him first line. This would be working in the opposite direction to his immunosuppression for his DM and would significantly risk worsening his DM. Cancer immunotherapy with checkpoint inhibitors is associated with frequent immune-related adverse events and is often not recommended for patients with concomitant autoimmune disease [7].

Discussions with the family and the patient took place around treatment. It was important not to push the patient too far with aggressive treatment. Equally, it was important not to wait too long for the patient to become too unwell to withstand even chemotherapy on account of deterioration from his DM. Prognosis calculation, in this situation, also proved difficult. The patient expressed his wish to be discharged home and to undergo trial chemotherapy on an outpatient basis. Unfortunately, he passed away about a week after discharge before any treatment could be formally commenced.

## Conclusions

While DM itself is a rare disorder, it is frequently associated with malignancy. DM is often treated with high-dose corticosteroids and immunomodulatory treatments. However, when DM is paraneoplastic, long-term disease control is frequently dependent on efficient therapy of the underlying cancer. In this example, a crucial therapeutic quandary arose due to the tumor's high PD-L1 expression, which would normally favor the use of immune checkpoint inhibitor (ICI) therapy. However, because this patient's DM remained clinically active and required high-dose immunosuppression, starting ICI therapy carried a larger risk of exacerbation. Chemotherapy was thus regarded as a safer initial treatment option. However, the patient's condition worsened before systemic anti-cancer treatment could begin. The take-home message from this case report is that timing is critically important in cases like these because the risk of rapid deterioration from DM will significantly and adversely impact cancer treatment options and considerations, necessitating the need for early multidisciplinary decision-making. This case also identifies a significant gap in knowledge about appropriate sequencing and safety measures for cancer immunotherapy in patients with active autoimmune illness.
